# Awareness of Stroke Risk Factors, Signs, and Symptoms Among Bahraini Adults Without Healthcare Training: A Cross-Sectional Survey

**DOI:** 10.7759/cureus.111591

**Published:** 2026-06-26

**Authors:** Khulood K Burashed, Ali J AlHilly, Yusuf J Hejres

**Affiliations:** 1 School of Medicine, Royal College of Surgeons in Ireland - Bahrain, Muharraq, BHR; 2 Neurology, Bahrain Defence Force Hospital - Royal Medical Services, Riffa, BHR; 3 General Practice, Salmaniya Medical Complex, Manama, BHR

**Keywords:** bahrain, population, risk, signs, stroke, symptoms

## Abstract

Background

Stroke is a leading cause of death and long-term disability worldwide, with incidence in Bahrain nearly doubling over the past 16 years. Early recognition of stroke risk factors and symptoms is critical for prevention and timely treatment. However, public awareness, particularly among individuals without a medical background, remains insufficiently studied in the Bahraini population.

Objective

This cross-sectional survey aimed to assess item-level recognition of selected stroke risk factors, warning signs, and symptoms among a convenience and snowball sample of adult residents of Bahrain without healthcare training. A secondary objective was to explore associations between educational attainment and correct recognition of individual questionnaire items.

Methods

A cross-sectional survey was conducted using an online questionnaire distributed through convenience and snowball sampling across Bahrain, yielding 1,564 responses. The questionnaire included demographic data, degree of knowledge of stroke risk factors, recognition of signs and symptoms, and degree of knowledge of actions to take when witnessing a stroke. Descriptive statistics were calculated for overall awareness, while Pearson’s chi-square and Cramér’s V tests assessed associations between education level and awareness.

Results

Smoking was the most widely recognized risk factor (91.3%), while inflammatory conditions (29.9%) and newborn age (30.5%) were the least recognized. Awareness of hypertension (76.6%), diabetes (63.1%), and atrial fibrillation (46.0%) demonstrated strong associations with education level (Cramér’s V = 0.350-0.549). Symptom recognition was highest for facial numbness (87.7%), whereas coordination issues (40.5%) and dizziness (45.7%) were poorly identified. Education significantly influenced awareness of most symptoms, with very strong associations for speaking difficulty (Cramér’s V = 0.497) and sudden confusion (Cramér’s V = 0.445). However, recognition of headache as a symptom of stroke decreased with higher education levels, suggesting misconceptions among more educated groups.

Conclusion

In this convenience and snowball sample of adults in Bahrain without healthcare training, awareness of stroke risk factors and symptoms varied substantially across educational levels, with notable gaps in recognition of several important risk factors and warning signs. Higher educational attainment was associated with greater awareness for most items; however, the cross-sectional design and unadjusted analyses preclude causal inference. These findings should be interpreted in light of potential recruitment bias and the use of a non-validated questionnaire, and they require confirmation in representative population-based surveys before informing national public health strategies.

## Introduction

Stroke is a medical emergency that occurs when the blood supply to the brain is disrupted, either due to an arterial blockage (ischemic stroke) or bleeding within or around the brain (hemorrhagic stroke). Globally, stroke is recognized as one of the leading causes of death and the foremost cause of long-term disability in adults, placing a significant burden on healthcare systems, families, and economies [1].

In Bahrain, the incidence of stroke has been increasing at an alarming rate, having almost doubled over the past 16 years [2]. A previous Bahraini study reported an incidence of approximately 110 per 100,000 population among Bahraini nationals in 2015, compared with approximately 57 per 100,000 in 1995 [2]. This trend is particularly concerning given the country’s relatively small population and the associated economic and social implications of rising disability rates.

Stroke survivors often face serious consequences that extend far beyond the acute event. Physically, they may experience muscle weakness, paralysis, and sensory disturbances, while cognitively, they may encounter difficulties with speech, language comprehension, and memory problems [3].

Timely recognition of stroke symptoms is critical for better outcomes. The widely promoted “time is brain” principle emphasizes that every minute without treatment results in the loss of millions of neurons. Intravenous thrombolytic therapy, using alteplase or, in selected patients, tenecteplase, remains a cornerstone of treatment when administered within the recommended therapeutic window. In addition, endovascular thrombectomy has become standard care for appropriately selected patients with large-vessel occlusion and can substantially improve functional outcomes when performed promptly. Many guidelines, such as in the study by Prabhakaran et al., require it to be administered within 4.5 hours from the onset of symptoms for maximal benefit [4]. Hence, delays in symptom/s recognition and seeking medical attention are among the leading barriers to receiving this life-saving treatment. Research shows that if thrombolysis is administered promptly, survival rates improve drastically, and an additional ~10% of patients can live independently after the event [5].

However, despite the potential for effective treatment, the best strategy remains prevention. Many strokes are considered preventable through the modification and effective management of well-established risk factors, including hypertension, diabetes mellitus, hyperlipidemia, smoking, obesity, and physical inactivity [6]. In Bahrain, the prevalence of these risk factors is notably high; for example, among stroke patients in one Bahrain study, ~75% had hypertension, ~54% had diabetes, and ~34% hyperlipidemia [2]. This high-risk profile is likely a major driver of the observed rising incidence of stroke in the country.

Public awareness of stroke encompasses several related but distinct domains. Awareness of stroke risk factors reflects knowledge of conditions and behaviors associated with increased stroke risk, whereas recognition of warning signs and symptoms relates to the ability to identify a possible stroke when it occurs. A third domain, intended emergency response, concerns the actions individuals would take upon witnessing stroke symptoms. Deficiencies in any of these areas may contribute to delays in prevention, recognition, and treatment. Early identification of stroke symptoms by patients, families, and bystanders is therefore essential for facilitating rapid activation of emergency medical services, timely hospital presentation, and access to evidence-based acute stroke therapies [7].

Studies from the Gulf Cooperation Council (GCC) countries and the wider Middle East and North Africa (MENA) region, for example, Kamran et al., have reported variable levels of public awareness regarding stroke risk factors and warning signs, with important knowledge gaps persisting despite ongoing public-health efforts [8]. However, contemporary data from Bahrain remain limited, particularly among adults without healthcare training and across different educational backgrounds. Furthermore, few studies, such as the one by Kamran et al., have examined how awareness varies according to educational attainment within the Bahraini population [8]. Therefore, this study aimed to assess awareness of stroke risk factors, signs, and symptoms among adults in Bahrain without healthcare training and to evaluate the association between educational attainment and stroke awareness. In addition, the study explored participants' intended responses when witnessing a potential stroke event [8].

## Materials and methods

Study design and setting

A cross-sectional survey was conducted in Bahrain between June 2024 and June 2025 to assess awareness of stroke risk factors, signs, and symptoms among adults without healthcare training. Participants were recruited through a combination of convenience and snowball sampling methods. The questionnaire was distributed electronically through social media platforms, and additional participants were recruited from individuals attending the neurology clinic at Bahrain Defence Force Hospital.

Participants

Eligible participants were adults residing in Bahrain who did not have a healthcare or medical background and who provided informed consent. For the purposes of this study, a healthcare or medical background was defined as employment in a healthcare profession, enrollment in a healthcare-related educational program, or formal medical or clinical training.

Participants were excluded if they were healthcare professionals, healthcare students, individuals with formal medical training, non-residents of Bahrain, declined consent, or submitted incomplete questionnaires.

Participants recruited from the neurology clinic were approached by members of the research team and were informed that participation was entirely voluntary. Treating physicians were not involved in obtaining consent or administering the questionnaire. Participants were informed that declining participation would not affect their medical care in any way. Survey responses were collected anonymously, and no personally identifiable information was recorded.

Questionnaire Development

The questionnaire was developed by the authors using educational material from the National Institutes of Health (NIH) related to stroke risk factors and warning signs. The instrument consisted of four sections containing closed-ended questions (Appendices). The questionnaire was not formally validated prior to administration.

The first section collected demographic information, including place of residence, age, whether participants are healthcare professionals, and highest educational attainment. It also assessed general knowledge of stroke, including prior awareness of stroke, personal history of stroke, perceptions regarding stroke treatability, knowledge of the organ affected by stroke, understanding of stroke occurrence across age groups, and awareness of stroke prevention.

The second section assessed awareness of stroke risk factors through questions with response options of 'Yes,' 'No,' and 'I do not know covering hypertension, diabetes mellitus, coronary heart disease, atrial fibrillation, heart valve disease, high low-density lipoprotein cholesterol, smoking, inflammatory conditions, neonatal age, and family history of stroke [9].

The third section assessed recognition of stroke signs and symptoms through questions with response options of 'Yes,' 'No,' and 'I do not know covering sudden numbness in the face, arm, and leg; sudden confusion; difficulty speaking/understanding speech; sudden trouble in vision or walking; dizziness; loss of balance; coordination issues; and sudden severe headache [10].

The final section assessed participants' intended response when witnessing an individual experiencing symptoms suggestive of stroke.

To reduce acquiescence bias, additional distractor items that were not established stroke risk factors or stroke symptoms were included within the questionnaire.

The survey was administered in both Arabic and English.

Data collection and consent

Participation was voluntary. Electronic informed consent was obtained before participants were allowed to access the questionnaire. Only questionnaires meeting the eligibility criteria and containing complete responses were included in the final analysis. No formal sample-size calculation was performed. Recruitment continued throughout the study period, yielding 1,564 eligible responses.

Data management

Responses were reviewed for eligibility and completeness before analysis. Incomplete questionnaires were excluded. No imputation of missing data was performed.

Outcome Measures

The primary outcomes were awareness of stroke risk factors and recognition of stroke signs and symptoms. Individual questionnaire items were analyzed separately. Correct identification of each risk factor and symptom was recorded as a positive response, and awareness was summarized as frequencies and percentages.

Statistical Analysis

Descriptive statistics were used to summarize participant characteristics and questionnaire responses. Frequencies and percentages were calculated for each risk factor and symptom overall and according to educational attainment.

Associations between educational level and awareness of individual risk factors and symptoms were evaluated using Pearson’s chi-square test. Cramér’s V was calculated to quantify the strength of statistically significant associations. Analyses were exploratory and unadjusted. Statistical significance was defined as a two-sided p-value of <0.05.

All findings should be interpreted in the context of the non-probability sampling design, which limits generalizability to the broader Bahraini population.

Given the exploratory nature of the study, multiple item-level comparisons were performed without formal adjustment for multiplicity. Therefore, individual p-values should be interpreted cautiously.

Ethical approval

Ethical approval was obtained from the Institutional Review Board of Royal Medical Services - Military Hospital on 14 December 2023 through the Crown Prince Centre for Training and Medical Research. Participation was voluntary, and electronic informed consent was obtained from all participants prior to survey completion. Survey responses were collected anonymously and were used solely for research purposes.

## Results

A total of 1576 responses were obtained. Twelve participants were excluded for not meeting the full inclusion criteria (7 non-residents and 5 below the age of 18).

**Figure 1:**
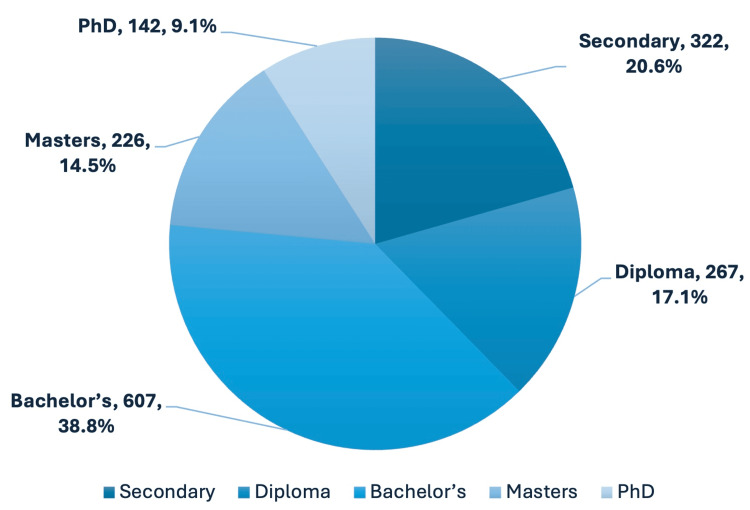
Participants’ demographic (level of education)

A total of 1564 of Bahrain’s residents were included in the study, with various levels of education. Demographics describing the level of education are shown in Figure 1.

Before assessing participants’ knowledge of stroke risk factors, signs, and symptoms, questions were asked regarding their personal history of stroke and their general awareness of stroke. These included awareness of what a stroke is, whether stroke is treatable, the body organ affected by stroke, understanding of stroke incidence, whether stroke can occur in younger individuals, and awareness of stroke prevention. The responses are summarized in Table 1.

**Table 1:** Participants’ basic understanding of strokes

Variable	Response	Frequency (n)	Percentage
Personal history of stroke	Yes	129	8.2%
	No	1435	91.8%
Awareness of stroke	Yes	1505	96.2%
	No	59	3.8%
Perception of stroke treatability	Yes	1162	74.3%
	No	164	10.5%
	I don’t know	238	15.2%
Knowledge of body organ affected by stroke	Brain	1423	91.0%
	Heart	134	8.6%
	Lungs	2	0.1%
	I don’t know	5	0.3%
Understanding of stroke incidence	Yes	994	63.6%
	No	185	11.8%
	I don’t know	385	24.6%
Can it occur in younger people	Yes	895	57.2%
	No	283	18.1%
	I don’t know	386	24.7%
Awareness of stroke prevention	Yes	856	54.7%
	No	115	7.4%
	I don’t know	593	37.9%

Awareness of risk factors

To begin extracting results and hence their analysis, we calculated the percentage of participants who correctly identified each stroke risk factor individually across the entire study population (shown in Table 2 under the “overall” column), which allowed us to highlight areas of both strength and weakness in public awareness. The highest level of recognition was for smoking, with 91.3%, n=1428 of participants identifying it correctly as a risk factor; this indicates that the community is already well-informed about the harmful effects of smoking on the brain's blood vessels and, hence, the rising stroke incidence. Therefore, compared to other factors, only a minimal additional public health education effort may be required in this area. In contrast, the lowest levels of awareness were observed for inflammatory conditions, recognized by only 29.9%, n=468 of participants; and newborns, which were identified as a risk factor by just 30.5%, n=477 of the sample. Hence, these findings suggest that substantial educational effort should be directed toward increasing awareness in these specific areas, as their lack of recognition indicates a significant related knowledge gap within the community.

**Table 2:** Risk factor awareness among people of varied educational backgrounds Data are expressed as % (n). Associations between educational attainment and risk factor awareness were assessed using Pearson's chi-square test. Cramér's V was calculated as a measure of association strength. Degrees of freedom (df) were calculated according to the dimensions of the contingency table. Analyses were exploratory and unadjusted for potential confounding variables. No formal adjustment for multiple comparisons was applied; therefore, p-values should be interpreted cautiously. Statistical analyses were performed using SPSS version 29.0.2.0 (IBM Corp., Armonk, NY, US). Confidence intervals were not calculated for individual item-level proportions.

Education	Secondary (n = 322)	Diploma (n = 267)	Bachelor’s (n = 607)	Masters (n = 226)	PhD (n = 142)	Overall (n= 1564)	χ²	P value	Cramér's V	Strength of Association
Risk Factor										
Hypertension	59.6% (192)	60.7% (162)	79.2% (481)	98.2% (222)	99.3% (141)	76.6% (1198)	191.68	<0.001	0.350	Relatively Strong
Diabetes	20.2% (65)	43.4% (116)	79.2% (481)	84.5% (191)	94.4% (134)	63.1% (987)	471.07	<0.001	0.549	Very Strong
CHD	27.6% (89)	55.1% (147)	77.8% (472)	83.6% (189)	98.6% (140)	66.3% (1037)	362.85	<0.001	0.482	Strong
Heart Valve Diseases	26.4% (85)	37.5% (100)	45.6% (277)	88.1% (199)	91.5% (130)	50.6% (791)	321.99	<0.001	0.454	Strong
Atrial Fibrillation	25.8% (83)	33.7% (90)	46.1% (280)	58.4% (132)	94.4% (134)	46.0% (719)	217.01	<0.001	0.373	Relatively Strong
High LDL Cholesterol	26.4% (85)	39.7% (106)	52.6% (319)	89.8% (203)	97.2% (138)	54.4% (851)	344.99	<0.001	0.470	Strong
Smoking	92.5% (298)	88.4% (236)	91.3% (554)	91.6% (207)	93.7% (133)	91.3% (1428)	4.50	0.342 NS	0.054	Negligible
Inflammatory Conditions	18.3% (59)	27.7% (74)	38.4% (233)	29.6% (67)	24.6% (35)	29.9% (468)	43.91	<0.001	0.168	Weak
Age (newborns)	14.3% (46)	24.0% (64)	26.0% (158)	43.8% (99)	77.5% (110)	30.5% (477)	217.67	<0.001	0.373	Relatively Strong
Family History	18.6% (60)	60.3% (161)	66.6% (404)	71.2% (161)	85.2% (121)	58.0% (907)	283.08	<0.001	0.425	Strong

Other risk factors that demand stronger public attention include hypertension (76.6%, n=1198), coronary heart disease (66.3%, n=1037), diabetes (63.1%, n=987), and high LDL cholesterol (54.4%, n=851). These conditions are not only prevalent within the Bahraini population but also carry a considerable potential for prevention through lifestyle modification and medical intervention, which makes it particularly concerning that related awareness is not uniformly high. In addition, atrial fibrillation, with an identification rate of only 46.0% (n=719), and heart valve disease, recognized by just 50.6% (n=791), remain underappreciated as contributors to stroke, even though they are clinically significant and should not be overlooked in education campaigns despite being less common than conditions such as hypertension or diabetes.

Finally, a family history of stroke, correctly identified by 58.0% (n=907) of participants, represents an interesting case because it is a non-modifiable risk factor. Nonetheless, raising awareness about it remains valuable, as individuals who recognize their inherent susceptibility are more likely to be vigilant about the early signs and symptoms of stroke, and this heightened awareness can ultimately lead to earlier medical attention and improved outcomes, even if direct prevention is not feasible.

Following our scanning and sorting of the entire participant pool, we extracted the data by education level. We then calculated chi-square and p values to determine statistical significance, along with Cramer's V test to measure the strength of association between stroke risk factors awareness and education level. The results (shown in Table 2) were as follows:

Awareness of hypertension as a stroke risk factor was strongly associated with higher education level. While only around 59.6% (n=192) of participants with secondary education and 60.7% (n=162) of diploma education recognized it, nearly all those with master’s and PhD degrees identified it correctly, reflecting a strong academic educational level effect.

Recognition of diabetes showed the most pronounced educational gradient. Awareness rose steeply from just 20.2% (n=65) among secondary-educated participants to 94.4% (n=134) among those with a PhD. The strength of association was very high, highlighting that knowledge of diabetes as a stroke risk factor is highly education-dependent.

A similar pattern was observed for coronary heart disease (CHD). Less than one-third of the secondary-educated group were aware of the role of CHD in stroke, compared with nearly universal recognition among PhD holders. This very strong association indicates that higher education greatly enhances understanding of cardiovascular comorbidities in relation to stroke.

Awareness of heart valve diseases also varied considerably by education level. Only about one-quarter of those with secondary education identified it as a risk factor (26.4%, n=85), compared to 91.5% (n=130) in the PhD group. This demonstrates a very strong educational influence and suggests that lower education groups may not fully appreciate the link between structural heart disease and stroke, and vice versa, for the higher education group.

For atrial fibrillation, awareness was low among the less educated, with only about one in four secondary-educated participants recognizing it, but this increased steadily with higher education level to 94.4% (n=134) among PhD-holders respondents. The strong association underscores the role of education in disseminating knowledge about rhythm disorders as risk factors for stroke.

A comparable trend was seen with high LDL cholesterol, where recognition ranged from 26.4% (n=85) in the secondary-educated group to 97.2% (n=138) in the PhD-holders group. This very strong association suggests that higher education substantially improves awareness of lipid-related risks.

In contrast, smoking was almost universally recognized as a stroke risk factor across all education level groups, with awareness levels consistently above 88%. There were no significant differences between participating groups, indicating that smoking is already well-established in the public’s understanding, regardless of education level.

Nevertheless, awareness of inflammatory conditions was low across all education groups, ranging from 18.3% (n=59) to 38.4% (n=233), with only modest variations. Although there was a statistically significant difference, the association was moderate, and even the most educated groups showed poor recognition of this risk factor. This highlights a significant gap in knowledge that requires targeted educational interventions.

In terms of age, awareness that newborns could also be affected by stroke increased with higher education, from 14.3% (n=46) in the secondary-educated group to 77.5% (n=110) among PhD holders. This strong association suggests that education level influences understanding of age-related risks, though overall awareness still lags compared to other, more common risk factors.

Finally, knowledge of family history followed a similar trajectory, with recognition rising from 18.6% (n=60) in secondary-educated participants to 85.2% (n=121) among PhD-holders respondents. This very strong association demonstrates that awareness of hereditary risk factors is heavily education-dependent.

Signs and symptoms awareness

After analyzing the results of stroke risk factor awareness, we proceeded to examine participants’ awareness of stroke signs and symptoms (shown in Table 3). The highest level of correct identification was found for face numbness, with 87.7% (n=1372) of total participants recognizing it as a symptom. Similar to smoking as a risk factor, this indicates that only minimal effort is needed to further strengthen public awareness in this area.

**Table 3:** Sign and symptom awareness among people of varied educational backgrounds Data are expressed as % (n). Associations between educational attainment and sign/symptom awareness were assessed using Pearson's chi-square test. Cramér's V was calculated as a measure of association strength. Degrees of freedom (df) were calculated according to the dimensions of the contingency table. Analyses were exploratory and unadjusted for potential confounding variables. No formal adjustment for multiple comparisons was applied; therefore, p-values should be interpreted cautiously. Statistical analyses were performed using SPSS version 29.0.2.0 (IBM Corp., Armonk, NY, US). Confidence intervals were not calculated for individual item-level proportions.

Education	Secondary (n = 322)	Diploma (n = 267)	Bachelor’s (n = 607)	Masters (n = 226)	PhD (n = 142)	Overall (n= 1564)	χ²	P value	Cramér's V	Strength of Association
Symptoms										
Face numbness	85.4% (275)	85.4% (228)	86.8% (527)	88.9% (201)	99.3% (141)	87.7% (1372)	21.38	<0.001	0.117	Weak
Arm numbness	58.7% (189)	76.8% (205)	81.5% (495)	81.9% (185)	91.5% (130)	77.0% (1204)	87.95	<0.001	0.237	Moderate
Leg Numbness	54.7% (176)	69.7% (186)	75.8% (460)	78.3% (177)	88.7% (126)	71.9% (1125)	77.14	<0.001	0.222	Moderate
Sudden confusion	25.5% (82)	42.7% (114)	68.0% (413)	77.4% (175)	95.8% (136)	58.8% (920)	310.24	<0.001	0.445	Strong
Difficulty speaking	27.6% (89)	59.6% (159)	76.3% (463)	92.9% (210)	97.2% (138)	67.7% (1059)	387.10	<0.001	0.497	Very Strong
Troubled vision	25.5% (82)	52.1% (139)	72.3% (439)	77.9% (176)	93.0% (132)	61.9% (968)	302.68	<0.001	0.440	Strong
Troubled walking	26.4% (85)	42.7% (114)	54.4% (330)	56.6% (128)	76.8% (109)	49.0% (766)	126.13	<0.001	0.284	Relatively Strong
Dizziness	23.0% (74)	44.6% (119)	47.0% (285)	59.7% (135)	71.0% (101)	45.7% (714)	122.45	<0.001	0.280	Relatively Strong
Loss of balance	26.1% (84)	45.3% (121)	65.4% (397)	74.8% (169)	89.4% (127)	57.4% (898)	248.50	<0.001	0.399	Strong
Coordination issues	18.3% (59)	36.7% (98)	56.0% (340)	36.7% (83)	37.3% (53)	40.5% (633)	129.89	<0.001	0.288	Relatively Strong
Headache	55.3% (178)	43.1% (115)	67.1% (407)	33.2% (75)	28.2% (40)	52.1% (815)	129.38	<0.001	0.288	Relatively Strong

Awareness of arm numbness (77.0%, n=1204) and leg numbness (71.9%, n=1125) was also relatively high. While the majority of participants correctly identified these symptoms, there is still room for improvement, and focused efforts could help enhance their recognition.

Moderate levels of awareness were noticed for sudden confusion (58.8%, n=920), speaking difficulty (67.7%, n=1059), troubled vision (61.9%, n=968), and loss of balance (57.4%, n=898). These findings highlight the need to reinforce education around these symptoms, as they are critical for the timely recognition of a stroke, but were only identified by less than two-thirds of participants.

The lowest levels of awareness were observed for coordination issues (40.5%, n=633), troubled walking (49.0%, n=766), dizziness (45.7%, n=714), and headaches (52.1%, n=815). Since less than half of the total participants identified these symptoms correctly, significant awareness campaigns are required to improve their recognition. These results emphasize the importance of targeted educational strategies to ensure that the population can identify a wide range of stroke symptoms, particularly the less obvious ones.

Awareness of face numbness as a stroke symptom was high across all educational groups, ranging from 85.4% (n=275) to 99.3% (n=141). Although the association with education was statistically significant, the strength was weak, indicating that recognition of this symptom is generally good regardless of education level.

In contrast, recognition of arm numbness showed a more distinct educational gradient, rising from 58.7% (n=189) in the secondary group to 91.5% (n=130) among PhD holders. The association was moderate, suggesting that higher education contributes to better awareness of this classic stroke symptom.

A similar trend was observed for leg numbness, with awareness increasing from 54.7% (n=176) among secondary participants to 88.7% (n=126) in the PhD group. The association was moderate in strength, showing that education plays an important role in recognizing motor-related symptoms of stroke.

Knowledge of sudden confusion was much lower among less educated groups (25.5%, n=82 in secondary) but increased steadily with education, reaching 95.8% (n=136) among PhD holders. The association was strong, highlighting that awareness of cognitive changes as stroke symptoms is heavily influenced by educational attainment.

For difficulty speaking, recognition improved dramatically with education: only 27.6% (n=89) of secondary participants identified it, compared with 97.2% (n=138) of PhD participants. This represents a very strong association, showing that education substantially enhances awareness of speech-related stroke symptoms.

Similarly, troubled vision was poorly recognized among secondary-educated participants (25.5%, n=82), but much better recognized by PhD holders (93%, n=132). The association was strong, confirming that visual symptoms are more likely to be identified by those with higher education.

Comparatively, awareness of troubled walking was low overall, starting at 26.4% (n=85) among secondary-educated participants and only reaching 76.8% (n=109) in the PhD-holders group. The association was relatively strong, suggesting that education was associated with greater awareness but that this symptom remains underrecognized compared to more typical stroke signs and symptoms.

Dizziness showed a similar pattern, with awareness levels ranging from 23% (n=74) in the secondary-educated group to 71% (n=101) in the PhD-holders group. The relatively strong association indicates that while education improves recognition, dizziness as a stroke symptom is still underappreciated by many.

Knowledge of loss of balance followed the same trajectory, rising from 26.1% (n=84) in the secondary-educated group to 89.4% (n=127) among PhD-holders participants. The strength of association was high, reflecting that education level strongly influences recognition of balance-related stroke symptoms.

For coordination issues, awareness levels increased from 18.3% (n=59) among those with secondary education to around 56% (n=340) among bachelor’s holders, but then declined again in the master’s and PhD holders groups (36-37%). The association was relatively strong, though the trend is irregular. The irregular distribution of responses across educational groups suggests that recognition of coordination issues as a stroke symptom may be influenced by variation in the interpretation of the questionnaire item. Because the survey did not explore participants' reasoning, no conclusions can be drawn regarding the underlying cause of this pattern.

Finally, awareness of headache as a stroke symptom varied inconsistently. It was relatively high among secondary-educated participants (55.3%, n=178) and bachelor’s holders (67.1%, n=407), but much lower among master’s (33.2%, n=75) and PhD holders (28.2%, n=40). The association was relatively strong, indicating significant differences across education groups, but in this case, higher education appears to decrease this symptom recognition. This may reflect greater awareness among highly educated individuals that headache is a less typical or nonspecific stroke symptom compared to others.

Intended response to suspected stroke

Participants were asked what action they would take if they witnessed an individual experiencing symptoms suggestive of a stroke. The majority indicated that they would call the emergency medical services number (999) (76.7%, n=1,199). A smaller proportion reported that they would personally transport the individual to the hospital (19.2%, n=301). Less appropriate responses were reported less frequently, including attempting resuscitation (3.1%, n=49) and sprinkling water on the person's face (1.0%, n=15).

These findings suggest that most participants recognized the need for urgent medical intervention in the event of a suspected stroke. However, approximately one-quarter of respondents selected alternatives to activating emergency medical services, highlighting potential gaps in emergency-response knowledge despite recognition of stroke risk factors and symptoms.

## Discussion

This cross-sectional survey of adults in Bahrain without healthcare training identified substantial variation in recognition of stroke risk factors and symptoms across educational groups within the recruited convenience and snowball sample. Recognition was generally higher for commonly recognized items, such as smoking and facial numbness, whereas several risk factors and symptoms demonstrated lower levels of recognition. Educational attainment was associated with recognition of many individual questionnaire items; however, given the observational cross-sectional design, non-probability sampling strategy, and unadjusted analyses, these findings should be interpreted as associations rather than evidence of causal relationships. Furthermore, the results reflect the characteristics of the recruited sample and should not be assumed to represent the broader Bahraini population.

The comprehensive survey of 1,564 individuals with varying education levels provides valuable insights into public awareness of stroke risk factors, signs, and symptoms. This discussion explores the implications of these findings, the influence of educational levels on awareness, and recommendations for targeted public health interventions.

Education and stroke knowledge: a clear relationship

The results showed, for most risk factors/signs/symptoms, a consistent and strong relationship between educational attainment and stroke awareness. This educational gradient is particularly pronounced for certain stroke symptoms, with PhD holders demonstrating significantly higher recognition rates compared to those with secondary education. This pattern aligns with previous research suggesting that higher educational achievement correlates with greater health literacy and disease awareness [11]. The strong associations observed for symptoms like speaking difficulty (Cramér's V = 0.497), sudden confusion (Cramér's V = 0.445), and troubled vision (Cramér's V = 0.440), shown in Table 3, suggest that education plays a pivotal role in recognizing the cognitive, motor, and sensory manifestations of stroke. This may reflect greater exposure to health education or enhanced ability to assimilate complex medical information among those with advanced educational degrees.

Disparities in symptoms/signs recognition

The findings reveal concerning disparities in symptom/sign recognition across education levels. For critical symptoms like speaking difficulty, recognition rates ranged dramatically from 27.6% (secondary education) to 97.2% (PhD), highlighting a troubling knowledge gap that could lead to delayed treatment-seeking among less-educated populations, as demonstrated in the results shown in Table 3. These disparities may contribute to documented inequalities in stroke outcomes across socioeconomic groups [12].

Interestingly, certain symptoms show relatively high recognition regardless of education, particularly face numbness (85.3-99.3% across all groups). This likely reflects the success of public health campaigns like FAST (Face, Arms, Speech, Time) that have emphasized facial drooping as a key stroke indicator [13]. The weak association (Cramér's V = 0.117), as demonstrated in Table 3, for this symptom suggests that effective public messaging can overcome educational barriers when properly implemented.

Unexpected findings and misconceptions

The irregular patterns observed for certain symptoms merit special attention. The recognition of coordination issues was unusually distributed, with bachelor’s degree holders showing higher awareness (56%) than both less educated groups and higher educated participants (master’s and PhD at 36-37%). This suggests the potential presence of misconceptions or conflicting information about this symptom, even among highly educated individuals.

Similarly, the inverse relationship between education and recognition of headache as a stroke symptom, where those with secondary education (55.3%) and bachelor’s degrees (67%) showed higher recognition than master’s (33.4%) and PhD holders (28.2%), may reflect greater understanding among highly educated participants that headache is a less specific stroke indicator [14]. This highlights the complex nature of health knowledge, where more education does not always translate to higher recognition rates for all symptoms, particularly those that are less distinctive or specific.

Implications for public health strategies

These findings have significant implications for stroke awareness campaigns and educational strategies. The clear educational gradient observed for most symptoms suggests that tailored approaches are needed for different educational demographics.

For less educated populations: Simplified, accessible messaging focusing on all stroke symptoms, with particular emphasis on less recognized indicators like sudden confusion, troubled vision, and coordination issues. Visual and narrative-based education materials may be more effective for these groups.

For moderately educated populations: More comprehensive information that builds on existing knowledge of classic symptoms while addressing specific gaps in awareness, particularly for symptoms like dizziness and troubled walking.

For highly educated populations: Clarification of more complex or ambiguous symptoms, addressing potential misconceptions about coordination issues and the role of headache in stroke presentation.

The findings also suggest that the successful messaging approach used for face numbness, which achieved high recognition across all education levels, could serve as a model for raising awareness of other symptoms. Adapting this approach to address less-recognized symptoms could potentially reduce the education-based disparity in stroke knowledge.

Limitations

This study has several limitations that should be acknowledged. The cross-sectional design limits the ability to establish a causal relationship between educational level and stroke awareness; therefore, the findings only reflect associations at a single point in time. The use of self-reported questionnaire responses may introduce response bias, as participants may overestimate their knowledge or select answers they believe are correct. The use of convenience and snowball sampling may limit the generalizability of the findings to the entire Bahraini population. Individuals with a greater interest in health-related topics or better access to online platforms may have been more likely to participate. Additionally, excluding individuals with a medical background helped focus the study on the general population, but it may also limit comparisons with healthcare-aware groups. Furthermore, education level may not fully represent health literacy, socioeconomic status, access to healthcare information, or personal exposure to stroke, all of which could influence awareness independently. The questionnaire was author-developed and not formally validated. Several risk-factor and symptom items may have been subject to differing interpretations, particularly those relating to neonatal stroke, inflammatory conditions, headache, dizziness, gait disturbance, and coordination difficulties. Consequently, some responses may reflect ambiguity in item wording rather than true differences in stroke knowledge. The analysis focused on recognition of predefined stroke risk factors and symptoms and did not incorporate a validated composite knowledge score. Although distractor items were included within the questionnaire, the primary analysis did not evaluate participants' ability to reject incorrect alternatives. Consequently, the findings should be interpreted as measures of item-specific recognition rather than comprehensive stroke knowledge. The analyses were unadjusted and did not account for potential confounding factors such as age, sex, socioeconomic status, prior exposure to stroke, language, or recruitment source. Consequently, the observed associations between educational attainment and awareness should not be interpreted as causal. Several questionnaire items may have been interpreted differently by participants because of ambiguity in terminology or varying familiarity with stroke-related concepts. This is particularly relevant for items relating to less specific symptoms and non-traditional risk factors. Because the questionnaire was author-developed and not formally validated, some responses may reflect differences in interpretation rather than true differences in knowledge.

Future directions

This study highlights several important directions for future research and public health practice.

Targeted interventions: Development and evaluation of educational interventions specifically designed for different educational demographics, with assessment of their impact on stroke knowledge and time to treatment.

Clarification of misconceptions: Research focusing on understanding the sources of confusion about symptoms like coordination issues and headache, particularly among highly educated groups.

Complementary approaches: Investigation of factors beyond education that may influence stroke awareness, such as media exposure, personal or family history of stroke, and access to healthcare.

Long-term impact assessment: Longitudinal studies to determine whether improvements in stroke symptom awareness translate to measurable reductions in treatment delays and improved outcomes.

An additional consideration for future stroke-awareness initiatives is the emergence of newer and potentially underrecognized risk factors, particularly among younger adults [15]. Electronic cigarette (e-cigarette) use has increased substantially worldwide and is increasingly being investigated for its potential cardiovascular and cerebrovascular effects [15]. Emerging evidence suggests that vaping may contribute to endothelial dysfunction, oxidative stress, inflammation, and thrombotic pathways that could increase stroke risk [15]. Similarly, illicit substance use, particularly stimulant drugs such as methamphetamine, has been associated with both ischemic and hemorrhagic stroke, often occurring at younger ages than traditionally observed in stroke populations [15]. Given increasing trends in e-cigarette use and substance misuse in several regions, including parts of Asia, future public-health campaigns and stroke-awareness studies should evaluate public knowledge of these emerging risk factors alongside established factors such as hypertension, diabetes, smoking, and atrial fibrillation. Incorporating these factors into future research may provide a more comprehensive understanding of stroke awareness and help identify evolving educational needs within the population [15].

## Conclusions

In this convenience and snowball sample of adults in Bahrain without healthcare training, recognition of stroke risk factors and symptoms varied across educational groups, with several items demonstrating lower levels of recognition than others. Because the study employed a non-probability sampling strategy, an author-developed non-validated questionnaire, and unadjusted analyses, the findings should be interpreted as exploratory and should not be assumed to represent the broader Bahraini population.

Future research should employ probability-based sampling methods and linguistically validated survey instruments to provide more representative assessments of stroke-related knowledge in Bahrain. Such studies should evaluate not only recognition of stroke risk factors and warning signs but also intended emergency responses to suspected stroke, as appropriate healthcare-seeking behavior is critical for timely treatment and improved outcomes. Further research is also needed to clarify awareness of emerging stroke risk factors, including electronic cigarette use and illicit stimulant use, particularly among younger adults.

## Appendices

Questionnaire

Introduction:

This research aims to determine the Bahraini population's awareness in regards to risk factors as well as signs and symptoms of stroke. That is in order to contribute to hopefully improve the outcome of stroke incidents by recognizing them early and as soon as they start.

Consent:

I agree to participate in this survey, as all my answers will be anonymous and my information will be treated confidentially.

**Table 4:** Stroke awareness in the population: survey Correct (keyed) responses are indicated by an asterisk (*). Distractor items were included to reduce acquiescence bias and assess participants' ability to distinguish established stroke-related items from incorrect alternatives.

Are you a resident/citizen of Bahrain?	Yes
	No
Are you above 18 years old?	Yes
	No
Do you work in the medical field?	Yes
	No
Level of education	Secondary
	Diploma
	Bachelor’s
	Masters
	PhD
Have you ever heard of a disease known as stroke?	Yes
	No
Have you ever had a stroke?	Yes
	No
A stroke occurs in the	Brain*
	Heart
	Lungs
	I don’t know
Can a stroke occur in young people?	Yes*
	No
	I don’t know
Incidence of stroke increases with age	True*
	False
	I don’t know
Do you think a stroke can be prevented?	Yes*
	No
	I don’t know
Stroke Risk Factors	
Is High Blood Pressure a risk factor of stroke	Yes*
	No
	I don’t know
Is Diabetes a risk factor of stroke	Yes*
	No
	I don’t know
Are Disease in the blood vessels of the heart risk factors of stroke	Yes*
	No
	I don’t know
Is abnormal electricity of the heart (atrial fibrillation) a risk factor of stroke	Yes*
	No
	I don’t know
Is heart valve disease a risk factor of stroke	Yes*
	No
	I don’t know
Is high LDL Cholesterol a risk factor of stroke	Yes*
	No
	I don’t know
Is smoking a risk factor of stroke	Yes*
	No
	I don’t know
Are Inflammatory conditions (viral illness) risk factors of stroke	Yes*
	No
	I don’t know
Are newborns at risk of stroke	Yes*
	No
	I don’t know
Are genetics a risk factor of stroke	Yes*
	No
	I don’t know
Is epilepsy a risk factor of stroke	Yes
	No*
	I don’t know
Are bowel diseases risk factors of stroke	Yes
	No*
	I don’t know
Is disc herniation in the back a risk factor of stroke	Yes
	No*
	I don’t know
Signs and symptoms: indicate whether each of the following is a sign/symptom of stroke	
Sudden numbness in the face	Yes*
	No
	I don’t know
Sudden numbness in the arm	Yes*
	No
	I don’t know
Sudden numbness in the leg	Yes*
	No
	I don’t know
Sudden confusion	Yes*
	No
	I don’t know
Difficulty speaking or understanding speech	Yes*
	No
	I don’t know
Sudden trouble in vision (seeing)	Yes*
	No
	I don’t know
Sudden trouble in walking	Yes*
	No
	I don’t know
Dizziness	Yes*
	No
	I don’t know
Loss of balance	Yes*
	No
	I don’t know
Coordination issues	Yes*
	No
	I don’t know
Sudden severe headache	Yes*
	No
	I don’t know
Back pain	Yes
	No*
	I don’t know
Shaking uncontrollably	Yes
	No*
	I don’t know
Abdominal pain	Yes
	No*
	I don’t know
Taking Action	
If you witness someone you think is having a stroke, what would you do?	Resuscitate
	Sprinkle water on their face
	Call 999 (an ambulance)*
	Drive them to the hospital on your own
